# Supplementary material for: Unveiling the Bioactive Potential of the Invasive Jellyfish Phyllorhiza punctata Through Integrative Transcriptomic and Proteomic Analyses
Source: Biomolecules. 2025 Aug 4;15(8):1121. doi: 10.3390/biom15081121 (PMC12383608; doi:10.3390/biom15081121)
Supplement: Supplementary file 1 [file biomolecules-15-01121-s001.zip › Suplementary Material/FileS3/PEPFOLD4/XP_065071966_17322/clusters.html]

 PEP-FOLD clusters


Download archive of all models  here   

|  |  |  |
| --- | --- | --- |
| # | file | sOPEP |
| # cluster 1 - Size: 1 |  |  |
|  | XP\_065071966\_17322-model1 | -5.61932 |
| # cluster 2 - Size: 20 |  |  |
|  | XP\_065071966\_17322-model2 | -5.43968 |
|  | XP\_065071966\_17322-model2.1 | -5.32285 |
|  | XP\_065071966\_17322-model2.2 | -5.16183 |
|  | XP\_065071966\_17322-model2.3 | -5.15508 |
|  | XP\_065071966\_17322-model2.4 | -5.13823 |
|  | XP\_065071966\_17322-model2.5 | -5.10098 |
|  | XP\_065071966\_17322-model2.6 | -5.09998 |
|  | XP\_065071966\_17322-model2.7 | -5.09158 |
|  | XP\_065071966\_17322-model2.8 | -5.06246 |
|  | XP\_065071966\_17322-model2.9 | -5.00893 |
|  | XP\_065071966\_17322-model2.10 | -5.00632 |
|  | XP\_065071966\_17322-model2.11 | -5.00005 |
|  | XP\_065071966\_17322-model2.12 | -4.98322 |
|  | XP\_065071966\_17322-model2.13 | -4.9786 |
|  | XP\_065071966\_17322-model2.14 | -4.97611 |
|  | XP\_065071966\_17322-model2.15 | -4.96549 |
|  | XP\_065071966\_17322-model2.16 | -4.96321 |
|  | XP\_065071966\_17322-model2.17 | -4.94514 |
|  | XP\_065071966\_17322-model2.18 | -4.9398 |
|  | XP\_065071966\_17322-model2.19 | -4.92119 |
| # cluster 3 - Size: 8 |  |  |
|  | XP\_065071966\_17322-model3 | -5.42851 |
|  | XP\_065071966\_17322-model3.1 | -5.38578 |
|  | XP\_065071966\_17322-model3.2 | -5.32216 |
|  | XP\_065071966\_17322-model3.3 | -5.26607 |
|  | XP\_065071966\_17322-model3.4 | -5.20894 |
|  | XP\_065071966\_17322-model3.5 | -5.17049 |
|  | XP\_065071966\_17322-model3.6 | -5.15334 |
|  | XP\_065071966\_17322-model3.7 | -5.0369 |
| # cluster 4 - Size: 6 |  |  |
|  | XP\_065071966\_17322-model4 | -5.42569 |
|  | XP\_065071966\_17322-model4.1 | -5.10544 |
|  | XP\_065071966\_17322-model4.2 | -5.08902 |
|  | XP\_065071966\_17322-model4.3 | -5.0508 |
|  | XP\_065071966\_17322-model4.4 | -5.04704 |
|  | XP\_065071966\_17322-model4.5 | -5.00737 |
| # cluster 5 - Size: 4 |  |  |
|  | XP\_065071966\_17322-model5 | -5.41935 |
|  | XP\_065071966\_17322-model5.1 | -5.40261 |
|  | XP\_065071966\_17322-model5.2 | -5.18271 |
|  | XP\_065071966\_17322-model5.3 | -4.9643 |
| # cluster 6 - Size: 3 |  |  |
|  | XP\_065071966\_17322-model6 | -5.41092 |
|  | XP\_065071966\_17322-model6.1 | -5.32883 |
|  | XP\_065071966\_17322-model6.2 | -5.27046 |
| # cluster 7 - Size: 6 |  |  |
|  | XP\_065071966\_17322-model7 | -5.31843 |
|  | XP\_065071966\_17322-model7.1 | -5.06273 |
|  | XP\_065071966\_17322-model7.2 | -4.96065 |
|  | XP\_065071966\_17322-model7.3 | -4.96015 |
|  | XP\_065071966\_17322-model7.4 | -4.94071 |
|  | XP\_065071966\_17322-model7.5 | -4.87232 |
| # cluster 8 - Size: 11 |  |  |
|  | XP\_065071966\_17322-model8 | -5.26775 |
|  | XP\_065071966\_17322-model8.1 | -5.26562 |
|  | XP\_065071966\_17322-model8.2 | -5.20785 |
|  | XP\_065071966\_17322-model8.3 | -5.15815 |
|  | XP\_065071966\_17322-model8.4 | -5.15066 |
|  | XP\_065071966\_17322-model8.5 | -5.11086 |
|  | XP\_065071966\_17322-model8.6 | -5.07668 |
|  | XP\_065071966\_17322-model8.7 | -5.07522 |
|  | XP\_065071966\_17322-model8.8 | -5.06579 |
|  | XP\_065071966\_17322-model8.9 | -4.98223 |
|  | XP\_065071966\_17322-model8.10 | -4.94259 |
| # cluster 9 - Size: 4 |  |  |
|  | XP\_065071966\_17322-model9 | -5.2667 |
|  | XP\_065071966\_17322-model9.1 | -5.20288 |
|  | XP\_065071966\_17322-model9.2 | -5.15446 |
|  | XP\_065071966\_17322-model9.3 | -5.11158 |
| # cluster 10 - Size: 8 |  |  |
|  | XP\_065071966\_17322-model10 | -5.26351 |
|  | XP\_065071966\_17322-model10.1 | -5.25384 |
|  | XP\_065071966\_17322-model10.2 | -5.17649 |
|  | XP\_065071966\_17322-model10.3 | -5.04749 |
|  | XP\_065071966\_17322-model10.4 | -4.99428 |
|  | XP\_065071966\_17322-model10.5 | -4.967 |
|  | XP\_065071966\_17322-model10.6 | -4.92793 |
|  | XP\_065071966\_17322-model10.7 | -4.90098 |
| # cluster 11 - Size: 2 |  |  |
|  | XP\_065071966\_17322-model11 | -5.25653 |
|  | XP\_065071966\_17322-model11.1 | -5.10426 |
| # cluster 12 - Size: 1 |  |  |
|  | XP\_065071966\_17322-model12 | -5.23631 |
| # cluster 13 - Size: 2 |  |  |
|  | XP\_065071966\_17322-model13 | -5.1082 |
|  | XP\_065071966\_17322-model13.1 | -5.022 |
| # cluster 14 - Size: 2 |  |  |
|  | XP\_065071966\_17322-model14 | -5.09783 |
|  | XP\_065071966\_17322-model14.1 | -5.03229 |
| # cluster 15 - Size: 7 |  |  |
|  | XP\_065071966\_17322-model15 | -5.05777 |
|  | XP\_065071966\_17322-model15.1 | -5.02459 |
|  | XP\_065071966\_17322-model15.2 | -5.01601 |
|  | XP\_065071966\_17322-model15.3 | -5.01117 |
|  | XP\_065071966\_17322-model15.4 | -5.00671 |
|  | XP\_065071966\_17322-model15.5 | -5.00425 |
|  | XP\_065071966\_17322-model15.6 | -4.96802 |
| # cluster 16 - Size: 2 |  |  |
|  | XP\_065071966\_17322-model16 | -5.0527 |
|  | XP\_065071966\_17322-model16.1 | -5.03389 |
| # cluster 17 - Size: 3 |  |  |
|  | XP\_065071966\_17322-model17 | -5.02111 |
|  | XP\_065071966\_17322-model17.1 | -4.90223 |
|  | XP\_065071966\_17322-model17.2 | -4.87552 |
| # cluster 18 - Size: 6 |  |  |
|  | XP\_065071966\_17322-model18 | -5.0004 |
|  | XP\_065071966\_17322-model18.1 | -4.9981 |
|  | XP\_065071966\_17322-model18.2 | -4.9981 |
|  | XP\_065071966\_17322-model18.3 | -4.99403 |
|  | XP\_065071966\_17322-model18.4 | -4.98564 |
|  | XP\_065071966\_17322-model18.5 | -4.87533 |
| # cluster 19 - Size: 1 |  |  |
|  | XP\_065071966\_17322-model19 | -4.98852 |
| # cluster 20 - Size: 1 |  |  |
|  | XP\_065071966\_17322-model20 | -4.9881 |
| # cluster 21 - Size: 2 |  |  |
|  | XP\_065071966\_17322-model21 | -4.91962 |
|  | XP\_065071966\_17322-model21.1 | -4.87552 |
|  |  |  |
